# Supplementary figures and images for: Loss of SP-A in the Lung Exacerbates Pulmonary Fibrosis
Source: Int J Mol Sci. 2022 May 10;23(10):5292. doi: 10.3390/ijms23105292 (PMC9141401; doi:10.3390/ijms23105292)

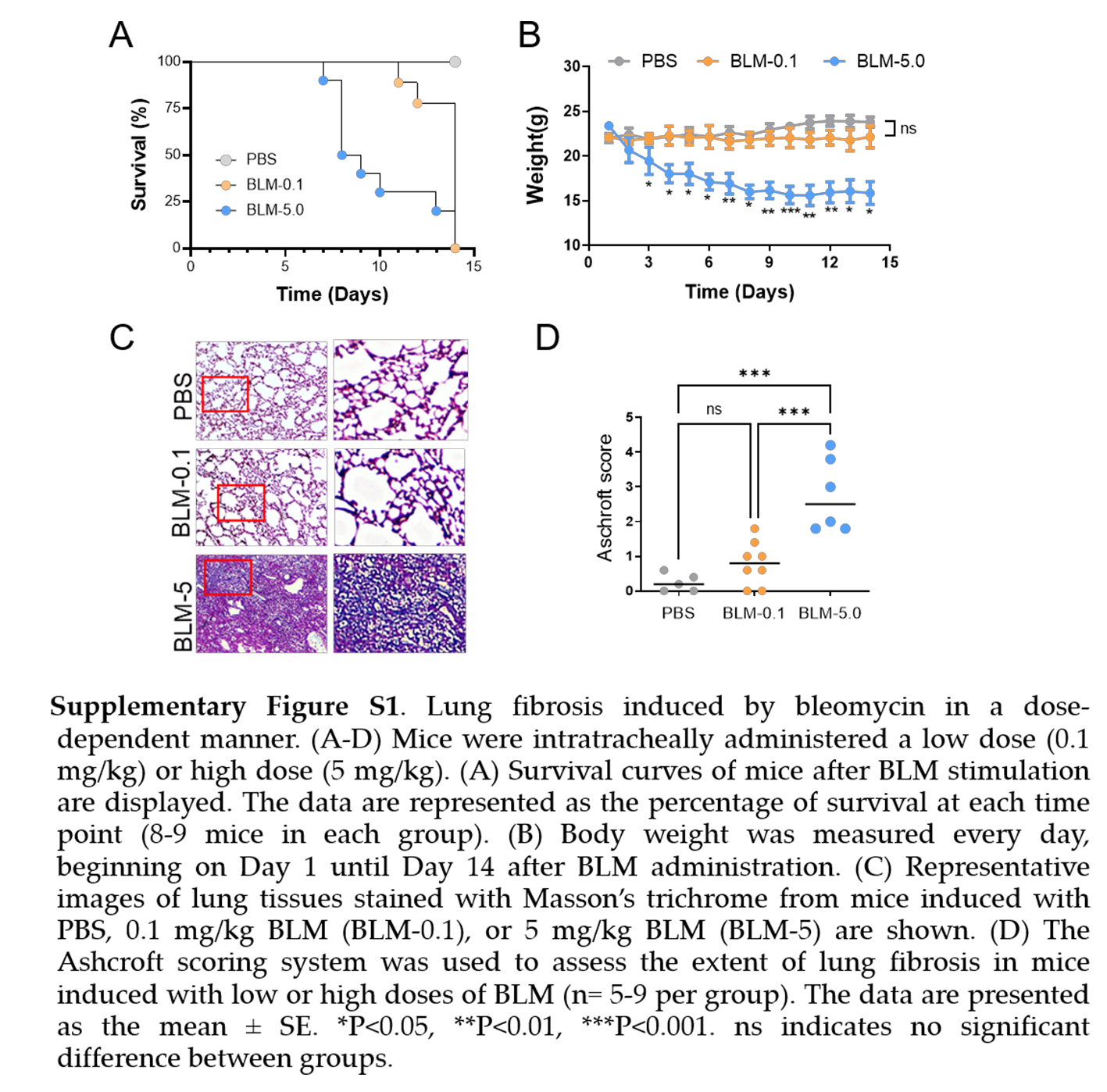

Supplement: Supplementary file 1 [file ijms-23-05292-s001.zip › Revised Suppl Figure.tif]
